# User-Centric Feedback for the Development and Review of a Unique Robotic Glove Prototype to Be Used in Therapy

**DOI:** 10.1155/2017/3896089

**Published:** 2017-06-04

**Authors:** Stuart James Biggar, Wei Yao, Lizhen Wang, Yubo Fan

**Affiliations:** ^1^Biomedical Engineering, University of Strathclyde, Wolfson Centre, 106 Rottenrow East, Glasgow G76 0HD, UK; ^2^School of Biological Science and Medical Engineering, Beihang University, Xueyuan Road No. 37, Haidian District, Beijing 100191, China

## Abstract

Disability can be a great impediment to daily living in later life and is often the result of illness or trauma. Modern thoughts on treatment are orientated towards the use of robotics; however, these are often designed without consultation with the user. This paper used a 5-point questionnaire to ask former therapy patients what they felt needed further improvements from potential robotics and what features of such a system were the most important. Significant emphasis was placed on helping them to grasp (*M* = 4.63) as well as having a functional use. They also desired a system with clearly distinguished (*M* = 4.22) and easy to operate controls (*M* = 4.44) whilst allowing them some freedom to move around independently (*M* = 4.44). This provided the rationale for a prototype dual-layered vacuum glove that was sampled by healthcare staff to provide feedback that forms the basis for future improvements.

## 1. Introduction

Improvements in technology have had a positive impact in many countries by increasing life expectancy. The downside of this is that it increases their chances of exposure to degenerative conditions; when this is coupled with poor lifestyle choices, it increases the possibility of impairment to their standard of living through disability [1, 2]. Increases in patient numbers means that health services must evaluate how best to adapt as the availability of staff to provide this therapy is reduced in both numbers and time [3, 4]. To bridge this gap, robotic systems can be utilized to enable therapy workers to attend to the needs of a greater number of patients. There are also potential benefits if the patient could use it in their own home to maximise exposure and the associated increase in exercise compliance [5, 6].

The first step in this process is to develop systems that work in conjunction with the therapists to help the patients to increase their recovery. The issue is that traditionally these devices will be developed to solve a problem that has been highlighted by the researcher, such as assisting mobility in structurally complicated joints like the shoulder [7] or the integration of design features for user feedback and control [8, 9], rather than a system that is developed in consultation with the patients. Whilst the designs perform their intended purpose, they can be large, bulky units that are not practical for a patient to take home, such as Dampace [10], while EMG controlled systems may also be challenging to set up and operate at home on their own due to a large number of electrodes requiring positioning to function [11]. Whilst a larger mechanism may be required for the shoulder due to its complexity, for hand-centric therapy, mechanical designs must accommodate the varied sizes of the fingers and be adaptable to this range and the changes in mechanical performance that result; an example of this is the adjustable iHandRehab system [12] that offers both active and passive control of the index finger and thumb. The design had a worn hand component that the user would interact with, along with a larger controller and actuator unit that is not worn; assessing the mobility of this device showed it to be achieving at least 86% of the maximal rotation in each joint of the thumb and 71.5% for the index finger. Smaller glove designs that are completely wearable have also begun to be developed, with examples such as the J-Glove and X-Glove [13, 14]. They are cable-driven designs that are portable and can be completely worn by the patient, although the results have not produced a significant improvement in patient outcomes, along with the design exposing the mechanism that enables its operation which may compromise the motion of the hand.

As a developing field, it is important to quantify the needs of the end user to ensure that the devices are used by patients. The best way to identify which areas patients are most in need of further support in their treatment is through discussion, although this lacks the formal structure to score the findings and assess trends. Pairing this with a questionnaire allowed for the formation of results in the assessment method Quality Function Deployment (QFD). This model originates in Japan and provides a reasoned process for how decisions can be reached by placing the needs of the user at the centre. The process begins with the product planning stage that highlights the design requirements and progresses to the characteristics of the parts to be used before outlining the planning and production phases for mass development. As a prototype is being developed in this study, only the initial phases are required. QFD has a pedigree of use in healthcare, such as diagnostic devices [15], cochlear implants [16], and power wheelchairs [17]. The purpose of this research was to consult with patients and use QFD to shape the design of a low-cost device that patients could use at home as both an assistive tool and as a therapy aid and to review it with therapy workers.

## 2. Method

The study consulted with former patients in central Scotland who were only eligible if their upper limb had been previously impaired and they had completed their prescribed therapy. The study discussed their experiences with this therapy to find out what they felt was and was not successful in their recovery. This was done through the combined use of a questionnaire and a follow-up interview to gather a mixture of qualitative and quantitative data to give the design features a weighted relevance to each other.

The questionnaire asked about five separate categories that had been highlighted to feature in the design (joint motion, function, control, wearability, and a combination of remaining aesthetic and practical features). These five categories had five criteria each of their own; the participants were asked to rank these criteria by importance in their opinion (five was to be considered the most important and one the least). To clarify this data, they were also asked to rank the whole categories to differentiate between criteria given the same rank across categories. Whilst the intention is to integrate as many of the 25 criteria into the design as possible, when these features clashed, the emphasis was placed towards the highest ranking criteria.

The initial data collection from the questionnaire was distributed to interested parties via physical activity groups, with participants being provided a copy of the studies paperwork and a stamped addressed envelope to return the questionnaire as well as the opt-in form for the interview. The interview was framed around their questionnaire responses, asking why the highest ranked features were the most important and why the lesser ones were not, using these points as a springboard for discussion over the merits of the features in the categories. Discussion of connections between the criteria that are in differing categories was encouraged to form a dialogue over the integration of functional needs in the overall design and allow the relationships to be quantified in the QFD design matrix.

**Figure 1:**
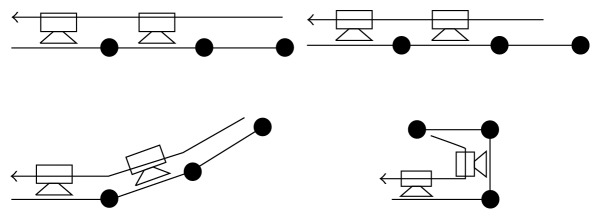
Illustration of process of finger rotation for glove design.

The information gathered in the matrix was used to create a design that developed into a prototype that was tested to get an indication of its performance in relation to its objectives. This prototype used a double-layered glove design that would grip tightly to the wearer by creating a vacuum within it, with suction cups being distributed on the inner layer. This resulted in the creation of fixed points within the glove that a cable could be threaded through to drive motion of the fingers (Figure 1); using these fixed points also results in the outer layer being used as a cover for the cables that prevents them from being impeded or damaged when items are grasped. The initially developed prototype was designed to assist with grasping, as this was highlighted as the main need of the former patients, but the principle could be reapplied to control finger extension, and the addition of a spring component would allow for both to be possible.

The use of the vacuum enables the skeleton of the wearer to be used as the frame for motion instead of an exoskeletal body, making it lighter than a metal frame and allows the device to be able to fit multiple wearers without needing components that are adjustable to fit each wearer due to variations in the length and width of their fingers. To further reduce the weight applied to the hand, the actuation system was placed on a separate unit on the forearm which would house the motors' mechanism and provide a set base for operation by attaching to the cables in the glove. The vacuum's outer layer also provides an additional protection to the cables from impact that is not provided to the exposed cables in previous designs [13, 14]. Whilst the creation of a vacuum can be loud and distracting, the aesthetic factors appearance and noise were amongst the lowest scoring criteria in the total study (1.78 and 1.56/5, resp.); consequently, they could be considered tolerable for the initial design.

**Figure 2:**
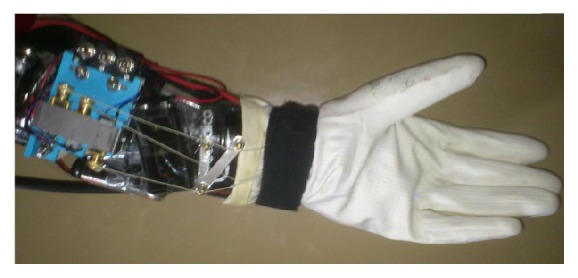
Photograph of developed prototype glove.

Applying the design matrix in conjunction with the design principles discussed resulted in the production of a functional prototype for user testing (Figure 2). The prototype replicated a pinch grasp with the cable-driven system and was a low-cost model using parts that were predominantly sourced locally. The layers were two brands of rubber gloves, with a braided fishing line used for the inner cable. Suction cups made from elastosil were threaded through the inner layer and were used as fixed points to connect the cable from the fingers to the motor. Activating the motors would shorten the length of the cable, creating rotation at the finger joints that would allow for replication of the motion of finger flexion. These motors were secured to a sports shin guard that provided a secure base on the forearm to distribute the weight and make the device easier to don and doff.

To control the device, a balance needed to be found between the number of commands that could be controlled and the ease with which those commands can be made; from the discussion, the prominent response was that it would be preferable to give the device fewer control options to lessen the effort required by the patient. Consequently, the motors that drive finger motion were controlled by a switch; this would be useable with orthopaedic injury population but would need to be revised in the future to make a device that was compatible with neurological conditions. The prototype structure has been trialled for its performance previously [18].

**Figure 3:**
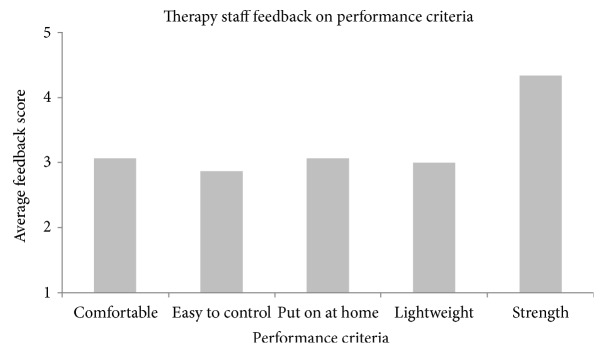
Graph of feedback scores for rehabilitation worker discussion.

Feedback was gathered from rehabilitation workers at the National Research Center for Rehabilitation Technical Aids in Beijing to get feedback from those who would be prescribing future devices to patients. They were asked to use their experience to provide feedback on how practical the device would be to use in treatment with their current patients and to provide input for future improvements to the design. 15 staff members (five rehabilitation doctors, four physical therapists, one occupational therapist, one orthopaedic surgeon, three orthotists, and one prosthetist) with an average experience of 6.4 years (SD = 5.25) in their role volunteered to sample the device and provide feedback on its function. They were given an opportunity to put on and try out the prototype to grasp and release large and small everyday items available in the department before discussing what they felt were the positives and negatives of the prototype; these included a pen, a telephone handset, a bottle of water, a watch, and a plastic tube. It was rated on the same five-point scale as was used with the unimpaired volunteers, and the average scores can be viewed in Figure 3. Each component of the study was approved by the local ethics committee and complied with the Declaration of Helsinki, with informed consent being received from the participants.

## 3. Results

37 former patients were approached at local physical activity groups to participate in the study, of which 13 responded to the questionnaire (response rate of 35.14%) with nine of those providing sufficient detail to be included in the study and eight of them agreeing to participate in the interview. The findings of the experimental process are shown in the matrix Table 1, where the results are firstly separated by their category and then further divided into their respective criteria. The results of the questionnaire are visible in the importance column and the relationship between the needs of the user and the critical functions having been made from the content of the interview discussions. The strength of the relationship is measured as a scale of 1, 3, and 9 where 1 indicates a weak relationship whilst 9 indicates a strong one. The rating of this relationship was judged based on the interview discussions conducted after the questionnaire, resulting from direct questioning, for example, how they felt that the discussed factors would impact on the level of control available to them, with the likelihood influencing the score assigned between the factors. An example of this is the wearability factors of comfort, weight, and stability all having a strong association with the idea of being easy to fit; therefore, to achieve this design requirement, all of the factors should be considered.

As illustrated in Table 2, a two tailed *t*-test was conducted to ascertain which criteria were significantly more important to the participants; however, the sample size means that the results may require scepticism. The *t*-test was used as the traditional means of assessing small data samples; Spearman's rho and Pearson's correlation require the data being assessed to be independent variables, where the ranking data gathered in this study is not. The *t*-test can still illustrate differences in the data however tends to produce more pronounced results; for example, hand grasping was thought to be most in need of further assistance post therapy in comparison to lifting (*r* = .035), rotation of the forearm (*r* = .00039), or reaching (*r* = .000015), whilst there was no significant difference to releasing (*r* = .1211). Additionally, in giving the user a sense of control over any possible system, it is important to ensure that the actions can be easily differentiated from one another (*M* = 4.22, SD = .667) as well as requiring as not requiring too much effort to operate (*M* = 4.44, SD = .726) to minimise fatigue. These results were utilised to design a wearable device that could be used for hand grasping by those with impaired mobility of their hand. A first-generation prototype was developed as can be seen in Figure 2 and was used for the sample testing.

Feedback on the prototype was provided by the rehabilitation workers, and the average score from their feedback can be viewed in Figure 3. They were asked to use their experience to consider how the device would perform with their patients. The workers believed that the prototype offered sufficient grip strength to be used as an assistive tool by patients (*M* = 4.33, SD = .617), although they had concerns about the comfort of the design and the level of control offered by the prototype (*M* = 3.07, SD = .799 and *M* = 2.87, SD = .64, resp.), the feedback from which will be used to improve the design in the future.

## 4. Discussion

The questionnaire showed that the most prevalent, but not significant, area of dissatisfaction with therapy was in the level of recovery achieved by the fingers (*M* = 3.78, SD = 1.481), in particular the act of grasping (*M* = 4.63, SD = .744), which aside from its related action of releasing was considered to be significantly more difficult than the arm-orientated actions of tilting and reaching (*r* = .0004 and .00001, resp.) with a difference to lifting being noticeable at the 95% confidence interval (*r* = .0355); therefore, it was best to focus the design on helping this action. Discussion with the former patients also made it clear that they were primarily hoping for a device that was able to assist them in the performance of tasks at home, although this perspective may come from the volunteers being at an advanced stage of their therapy. Consequently, ensuring that the designed prototype could perform this action was the key outcome of the design stage.

Having defined the central activity of the device, consideration must be given to the method of control. In the opinion of these former patients, the most important factors to giving them control over the device would be to minimise the effort required to make commands as well as how easily they can differentiate between them (*M* = 4.44, SD = .726 and *M* = 4.22, SD = .667, resp.). To accommodate these criteria, the options for control would need to be minimised as increased movement options will cause a conflation of the commands and reduce the control offered to the user. Therefore, to best meet the dominant functional and control criteria, the motion of the fingers should be limited to flexion and extension axis for control of grasping, as this motion can be controlled by a linear system such as a switch.

When designing a device that can be used at home by patients, how comfortable the device is to wear is a core consideration that should be considered alongside its function and control. The system's weight was deemed an important factor in how wearable any device is (*M* = 4, SD = .866) and has an association with its lifespan and ease of fitting; these factors are also important for giving the wearer freedom to move around as well as the setup time. Minimising the weight can also help meet the patients' needs in other factors, and it is also important to consider how comfortable any design is (*M* = 3.67, SD = 1.581); both of these factors scored higher in their category than the other features, but not significantly, but the ideas have been successfully applied in the past with the lower limb soft exosuit [19]. Less emphasis was placed on the stability of the components, although this may result in inconsistency in the device's performance. With upper limb disability of patients, particularly if their injury also weakens their shoulder, it becomes increasingly important to make the device as lightweight as possible to enable them to utilise it as they are moving.

Beyond these factors, the most prominent design considerations were that the participants hoped to retain the freedom of movement whilst using the device as well as it ensuring their safety (*M* = 4.44, SD = .527 and *M* = 4.33, SD = .707, resp., with significant differences between these 2 factors and those remaining in the category at a 95% confidence interval). Some previous designs of therapeutic devices require to be fixed in position to operate [20, 21], which whilst beneficial to recovery do not allow for use in an assistive capacity. The main anomaly from this result is that the respondents placed the importance of functionality over their own safety which may be due to a combination of factors: firstly, the volunteers for this study were at an advanced stage of recovery than patients who would be using the device are and this may distort their expectations for what can be achieved; additionally, it is possible that the participants were unaware of the risks that may occur in terms of further damage if the device is not properly designed, which is partly supported by over half of the volunteers considering the mobility allowed to be more important than the protections it provides them, but due to the importance of protecting the patient in therapy, this factor should remain a key consideration.

This provided the basis for the design that was outlined previously to develop a prototype that could be used for preliminary sampling to improve further. When sampling the device, the rehabilitation workers were able to securely grasp the objects and were then able to use them, as intended, such as the telephone and the pen for writing. The only exception to this was grasping a bottle of water, where the material compresses under the pressure exerted by the device, making the grasp unstable. The rehabilitative workers scored the strength of the device at *M* = 4.33, SD = 0.617; the scoring of this factor would suggest that the principle of design is viable for use with a patient population as an assistive tool for daily living, but will require further refinement in other areas of design.

The comfort of the device was considered to be another area that required improvement in the opinion of the rehabilitation professionals (*M* = 3.067, SD = .799). These areas were firstly that there is a temperature buildup that occurs over prolonged wearing, and this resulted in sweating that made removing the rubber gloves more challenging. Secondly, the size and weight of the forearm unit was considered to be too large, although this issue was reported by the smallest volunteers, suggesting that the size issue may be remedied by appropriately scaling the component to the wearer, although reductions must be made to the total weight of 525 grams before it could be considered suitable for patient use. This is 125 grams heavier than the similarly sized SaeboFlex. Reductions in the weight of these components are possible in future iterations of the design to make it more comfortable to wear, and this can be achieved by optimising the components distributed on the forearm.

The ease of control that the prototype offers to the patients was also considered to require improvements by the rehabilitation workers (*M* = 2.867, SD = .64). This was due to their expressed concern that patient's may overfocus on managing the cable mechanism rather than observing the motion of their fingers; this may result in the fingers being overstrained and risks harming them, a flaw that could be amended with the addition of an automatic brake. There were also concerns raised about the 3 control switches used to operate the fingers, firstly that the number of switches may be confusing for a patient who has also experienced a mental injury and secondly that they may be too stiff for a weakened patient to operate. The switches could be replaced in future designs with alternatives that enable a singular control for activity, or allow the patient to operate it with a mechanism that supports neurorehabilitative recovery. The variety of control mechanisms used with the J-Glove [13] shows the possibilities for control that can be achieved without burdening the patient.

## 5. Conclusion

This study has developed a low-cost robotic prototype intended for disability patients that can be used for rehabilitation as well as being used as an assistive tool at home. It was designed in accordance with the stated requirements of former therapy patients from a combined questionnaire and interview that was then integrated into a matrix and used to build a working prototype. This prototype was sampled with a group of medical workers to gather their feedback on what the strengths and weaknesses of the design were and to discuss further refinements that could be made to the prototype to improve it in the future.

## Figures and Tables

**Table 1:** First design matrix table connecting the user's needs to the critical functions of the product.

Category	Design criteria	Importance (ranking of 1–5)	Range of motion	Ease of control	Consistency of response	Independence	Long life	Ease of fitting	User protection	Low cost	How much?
Joint motion Category importance: 4	Shoulder	2.56	9	3	9	9	9	3	3	3	5 dof
	Elbow	2.89	9	3	9	9	3	3	3	9	2 dof
	Wrist	2.67	9	3	9	9	3	3	3	3	3 dof
	Fingers	3.78	9	3	9	9	9	3	3	9	4 dof
	Thumb opposability	3.11	9	3	9	9	9	3	3	9	3 dof
Function Category importance: 4.78	Grasping	4.63	3	9	9	9	9	3	3	9	Clinical evaluation
	Lifting	3	9	9	9	9	9	3	9	3	Clinical evaluation
	Releasing	3.63	3	9	9	9	9	3	1	9	Clinical evaluation
	Tilting/rotation	2.25	9	9	9	9	3	3	3	3	Clinical evaluation
	Reaching	1.5	9	9	9	9	3	3	1	3	Clinical evaluation
Interaction /control Category importance: 2.67	Ease of selection	4.22	9	9	9	9	9	9	3	3	Testing
	Starting motion	2.56	3	9	9	9	3	1	3	9	Testing
	Stopping motion	1.44	3	9	9	9	3	1	9	9	Testing
	Effort	4.44	3	9	9	9	9	1	3	3	Clinical evaluation
	Feedback	2.33	1	1	9	3	9	1	9	3	Haptic devices
Wearability Category importance: 2.22	Comfort	3.67	3	1	3	3	9	9	3	3	User feedback
	Weight	4	3	3	1	9	9	9	9	3	<1 kg
	Stability	2.33	9	1	3	3	9	9	9	3	Clinical evaluation
	Tightness at joints (fit 1)	2.33	3	1	3	3	9	9	3	9	Clinical evaluation
	Tightness at muscles (fit 2)	2.67	3	1	3	3	9	9	3	9	Clinical evaluation
Other Category importance: 1.33	Setup	2.89	3	3	1	9	9	9	9	9	Market analysis
	Appearance	1.78	9	1	1	3	9	1	1	3	User feedback
	Noise	1.56	1	1	1	3	9	1	1	3	User feedback
	Freedom	4.44	9	9	9	9	9	9	9	3	Clinical evaluation
	User safety	4.33	9	9	9	9	9	9	9	9	Clinical evaluation

The matrix depicts the strength of relationship between factors on a 3-point scale where 1 is a weak correlation and 9 is a strong correlation. The relationship strength was taken from the participant interviews after the questionnaire. dof stands for degrees of freedom.

**Table 2:** Two tailed *t*-test of questionnaire scores.

	Importance	Joint motion	Function	Interaction	Wearability	Other
Joint motion	4	N/A	.06528478	.02850921	.00058196^∗^	.00001529^∗^
Function	4.78	.06528478	N/A	.00032041^∗^	.00006532^∗^	.00000005^∗^
Interaction	2.67	.02850921	.00032041^∗^	N/A	.44681333	.01142455
Wearability	2.22	.00058196^∗^	.00006532^∗^	.44681333	N/A	.05160895
Other	1.33	.00001529^∗^	.00000005^∗^	.01142455	.05160895	N/A
Joint motion		Shoulder	Elbow	Wrist	Fingers	Thumb
Shoulder	2.56	N/A	.63053608	.89606922	.23611504	.50770165
Elbow	2.89	.63053608	N/A	.75992297	.29290489	.75314164
Wrist	2.67	.89606922	.75992297	N/A	.14911670	.57763523
Fingers	3.78	.23611504	.29290489	.14911670	N/A	.28153692
Opposable thumb	3.11	.50770165	.75314164	.57763523	.28153692	N/A
Function		Grasping	Lifting	Releasing	Tilting/rotation	Reaching
Grasping	4.625	N/A	.03542516	.12112229	.00039143^∗^	.00001463^∗^
Lifting	3	.03542516	N/A	.40509395	.22160142	.02628739
Releasing	3.625	.12112229	.40509395	N/A	.05434357	.00612321^∗^
Tilting/rotation	2.25	.00039143^∗^	.22160142	.05434357	N/A	.19702207
Reaching	1.5	.00001463^∗^	.02628739	.00612321^∗^	.19702207	N/A
Interaction		EoS	Start	Stop	Effort	Feedback
Ease of selection	4.22	N/A	.00352202^∗^	.00002641^∗^	.59426402^∗^	.00454422^∗^
Starting motion	2.56	.00352202^∗^	N/A	.04035065	.00066491^∗^	.71883630
Stopping motion	1.44	.00002641^∗^	.04035065	N/A	.00000636^∗^	.15355473
Effort	4.44	.59426402	.00066491^∗^	.00000636^∗^	N/A	.00707767^∗^
Feedback	2.33	.00454422^∗^	.71883630	.15355473	.00707767^∗^	N/A
Wearability		Comfort	Weight	Stability	Fit 1	Fit 2
Comfort	3.67	N/A	.56319426	.14111328	.10378649	.30520137
Weight	4	.56319426	N/A	.02416573	.00539088^∗^	.09607159
Stability	2.33	.14111328	.02416573	N/A	1.00000000	.61954375
Fit 1—joints	2.33	.10378649	.00539088^∗^	1.00000000	N/A	.63053608
Fit 2—muscles	2.67	.30520137	.09607159	.61954375	.63053608	N/A
Other		Setup	App	Noise	Freedom	Safety
Setup	2.89	N/A	.06188556	.02220390	.00542273^∗^	.02602469
Appearance	1.78	.06188556	N/A	.59426402	.00004367^∗^	.00000049^∗^
Noise	1.56	.02220390	.59426402	N/A	.00000391^∗^	.00012037^∗^
Freedom	4.44	.00542273^∗^	.00004367^∗^	.00000391^∗^	N/A	.75992297
User safety	4.33	.02602469	.00000049^∗^	.00012037^∗^	.75992297	N/A

The table shows the value of a 2-tailed *t*-test comparing the factors of the questionnaire within their category. A significant relationship (*p* ≤ .01) is denoted with ∗. EoS is ease of selection.
